# Phenylethanoid Glycosides from *Cistanche tubulosa* (Schenk) Wight Improve Radiation-Induced Bone Injury and Reduce Grem1 Expression in Osteoblasts

**DOI:** 10.3390/ijms27094109

**Published:** 2026-05-04

**Authors:** Xiaoqiang Wang, Xianxie Zhang, Yi Ru, Wenrun Yu, Pengfei Du, Ao Kong, Liping Chen, Zengchun Ma, Yuguang Wang

**Affiliations:** 1 Institute of Traditional Chinese Medicine, Tianjin University of Traditional Chinese Medicine, Tianjin 301617, China; xiaoqiangw1997@163.com (X.W.); yuwenrun035@163.com (W.Y.); dpflos@163.com (P.D.); 2 Academy of Military Medical Sciences, Beijing 100850, China; zhangxianxie@163.com (X.Z.); ruyi9809@163.com (Y.R.); kongao17839448007@163.com (A.K.); clp157707503482022@163.com (L.C.); mazchun@139.com (Z.M.)

**Keywords:** radiation-induced bone injury, bone loss, *Cistanche tubulosa*, single-cell RNA sequencing, Grem1

## Abstract

Radiation-induced bone injury (RIBI) is a delayed complication of radiotherapy that causes progressive bone loss and impairs skeletal integrity. In this study, we investigated whether phenylethanoid glycosides (PGs) from *Cistanche tubulosa* (Schenk) Wight could improve RIBI and explored the underlying mechanism. Using a mouse model of delayed radiation-induced bone injury, we found that PGs improved body weight recovery, grip strength, and oxidative stress status after irradiation. Through micro-computed tomography and histological staining to evaluate bone microstructure, it was found that PGs could reduce osteoclast activity after radiation and restore trabecular microstructure, thereby alleviating bone loss caused by radiation. Single-cell RNA sequencing of femoral bone marrow revealed that radiation disrupted the cellular composition of the bone marrow microenvironment and impaired osteogenic programs in bone marrow mesenchymal stem cells. PGs partially restored osteogenic differentiation-related signatures and suppressed adipogenic bias. In osteoblasts, we identified Gremlin-1 (Grem1), a negative regulator of BMP signaling, as a key radiation-responsive gene that was markedly upregulated after irradiation and downregulated by PGs. Western blotting in femoral tissue and MC3T3-E1 cells confirmed that PGs suppressed radiation-induced Grem1 expression. In vitro, PGs promoted alkaline phosphatase activity and mineralization in irradiated osteoblasts. These findings suggest that PGs mitigate RIBI by attenuating oxidative stress, improving bone remodeling, restoring the bone marrow microenvironment, and relieving Grem1-mediated inhibition of osteogenesis. Grem1 may represent a potential therapeutic target for radiation-associated skeletal injury.

## 1. Introduction

Radiotherapy is a key component of modern cancer treatment, with more than 50% of patients receiving radiation during therapy [1]. As tumor control and survival improve, late adverse effects of radiotherapy have become an increasing clinical concern. Radiation-induced bone injury (RIBI) is a delayed complication of radiotherapy characterized by progressive bone loss, decreased bone mineral density, and increased risk of pathologic fracture, which significantly affects the long-term quality of life of patients undergoing radiotherapy [2]. Despite its clinical relevance, effective approaches to prevent or reverse RIBI remain limited, and the underlying mechanisms are still incompletely understood.

Radiation disrupts skeletal homeostasis through several processes, including oxidative stress, chronic inflammation, changes in the bone marrow microenvironment, and dysfunction of mesenchymal stem and progenitor cells. Importantly, even when irradiated bone appears morphologically repaired, persistent microarchitectural abnormalities and altered cellular composition are frequently observed [3,4,5], suggesting long-lasting impairment of bone remodeling. However, the detailed cellular landscape of the post-irradiation bone marrow niche, as well as the molecular regulators responsible for defective osteogenesis, remain unclear.

Phenylethanol glycosides (PGs) are the main active substances of *Cistanche tubulosa* (Schenk) Wight (Orobanchaceae). They possess polyphenolic structures containing ortho-dihydroxyphenyl (catechol) moieties. Among them, echinacoside (C_35_H_46_O_20_) and acteoside (C_29_H_36_O_15_) are the key active monomeric compounds with antioxidant, anti-inflammatory, and tissue-protective properties [6,7,8]. Previous studies have shown that PGs enhance stem cell proliferation and osteogenic differentiation through pathways such as Wnt/β-catenin, Nrf2, and PI3K/Akt and improve disorders of bone metabolism [9,10]. However, their potential role in radiation-induced bone injury and the associated mechanisms remain largely unexplored.

Recent advances in single-cell RNA sequencing (scRNA-seq) have enabled high-resolution analysis of cellular heterogeneity and transcriptional changes in complex tissues under radiation stress. Emerging evidence indicates that stromal, hematopoietic, and immune cell populations exhibit distinct radiation-responsive programs and that the regenerative capacity of specific progenitor subsets is compromised at late time points after irradiation [11,12].

In this study, we used our previously established mouse model of delayed radiation-induced bone injury [13]. Using this model, we were able to mimic bone damage after radiotherapy and explore the effects of PGs following irradiation. By integrating micro-CT, histological analyses, and scRNA-seq of the bone marrow microenvironment, we characterized radiation-induced changes in bone remodeling and cellular composition. We further found that Grem1 is aberrantly upregulated in post-irradiation osteoblasts and that PGs inhibit the aberrant upregulation of Grem1.

## 2. Results

### 2.1. PGs Improve Physiological Status in Mice with RIBI

**Figure 1:**
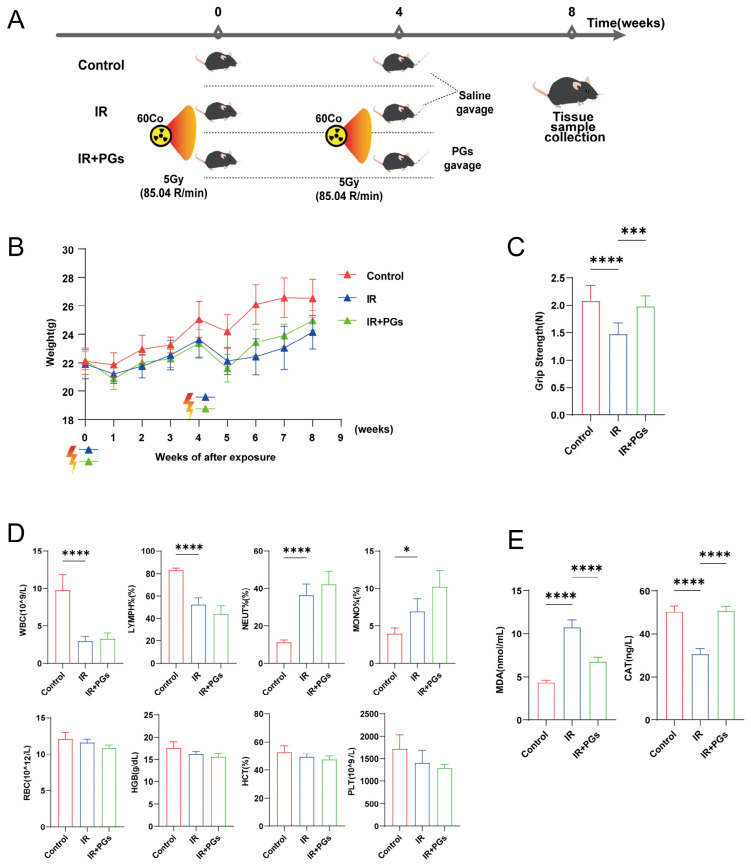
Intervention of PGs on radiation-injured mice. (**A**) Schematic of animal grouping and treatment procedures. Mice in the IR and IR + PGs groups received two rounds of total body irradiation (total dose: 10 Gy) spaced four weeks apart, while the Control group received no irradiation. Starting after the second irradiation, the IR + PGs group was administered PGs (150 mg/kg/day) by oral gavage, and the Control and IR groups received equal volumes of saline. (**B**) Body weight changes of mice over time (*n* = 10). Radiation-induced body weight loss at one-week post-irradiation was gradually recovered, a process accelerated by PGs treatment. (**C**) Forelimb grip strength of mice (*n* = 10). PGs treatment promoted the recovery of grip strength impaired by irradiation. (**D**) Peripheral blood cell counts (*n* = 6). Irradiation induced marked dysregulation in the populations of major immune cell types. (**E**) Systemic oxidative stress levels (*n* = 6), as measured by MDA content and CAT levels. Data are presented as mean ± SD. * *p* < 0.05, *** *p* < 0.001, **** *p* < 0.0001.

We used our previously established mouse model simulating radiological bone injury after radiotherapy and evaluated the role of PGs after irradiation (Figure 1A). Compared with the Control group, mice in the IR and IR + PGs groups exhibited significant body weight loss one week after the second irradiation, indicating substantial physiological impact of radiation. After PGs intervention, mice in the IR + PGs group showed a significant recovery trend in body weight compared with the IR group (Figure 1B). Grip strength testing (Figure 1C) showed that mice in the IR group had significantly reduced grip strength compared with the Control group (*p* < 0.0001), suggesting impaired bone integrity. PGs treatment significantly increased grip strength compared with the IR group (*p* < 0.001), reaching levels close to those of the Control group.

Whole blood analysis (Figure 1D) showed continued impairment of the immune system after 4 weeks of secondary irradiation. Compared with the Control group, total white blood cell counts were significantly reduced (*p* < 0.0001), with decreased lymphocyte proportion and increased neutrophil (*p* < 0.0001) and monocyte (*p* < 0.05) proportions. Red blood cell indices showed a decreasing trend without significant differences, suggesting impaired erythropoiesis. PGs intervention did not significantly restore hematological indices at this dose, indicating limited direct effects on hematopoietic recovery.

Serum assays revealed that CAT levels were significantly decreased and MDA levels significantly increased in the IR group (*p* < 0.0001), indicating enhanced oxidative stress (Figure 1E). PGs treatment significantly improved these oxidative stress markers, suggesting an antioxidant protective effect.

### 2.2. PGs Reduce Bone Loss in Mice with RIBI

**Figure 2:**
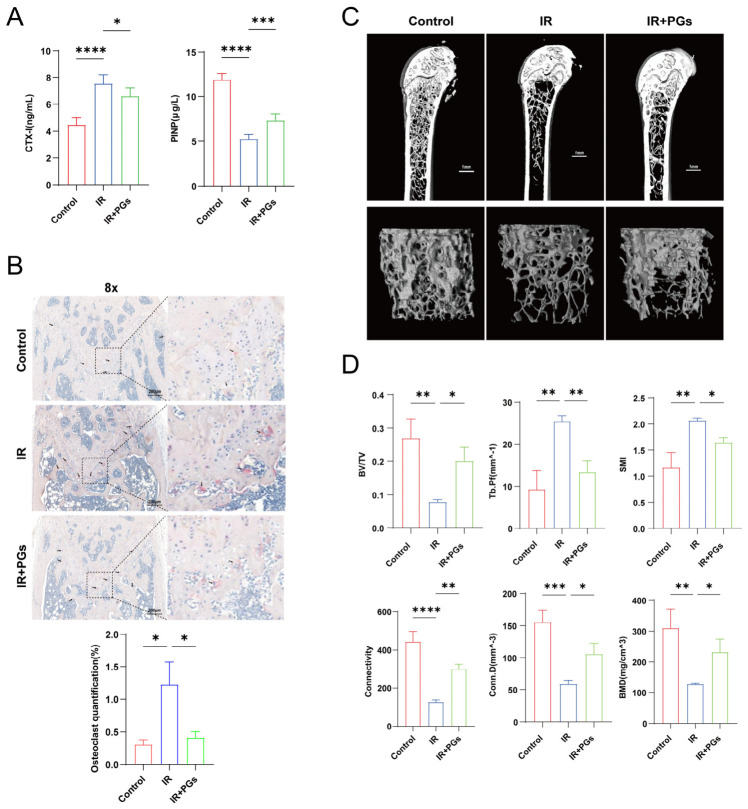
PGs promote the restoration of bone microstructure and bone tissue in irradiated mice. (**A**) Serum levels of bone turnover markers (*n* = 6). Irradiation significantly elevated the bone resorption marker CTX-I and reduced the bone formation marker PINP. These changes were effectively reversed by PGs intervention. (**B**) Representative TRAP-stained sections of femoral trabecular bone and quantitative analysis of osteoclast surface (*n* = 3). (**C**) Representative 3D micro-CT reconstruction images of distal femoral metaphysis (*n* = 3). The IR group exhibited a notable loss in trabecular bone, which was ameliorated by PGs treatment. (**D**) Quantitative micro-CT analysis of trabecular bone. A 1.5 mm long region starting 0.5 mm below the growth plate was analyzed. Parameters include BV/TV (bone volume fraction), Connectivity (trabecular connectivity), Conn.D (connectivity density), BMD (bone mineral density), SMI (structure model index), and Tb. Pf (trabecular pattern factor) (*n* = 3). Data are presented as mean ± SD. * *p* < 0.05, ** *p* < 0.01, *** *p* < 0.001, **** *p* < 0.0001.

We used enzyme-linked immunosorbent assay (ELISA) to detect the contents of type I collagen C-terminal cross-linked terminal peptide (CTX-I) and type I pron-terminal propeptide (PINP), respectively (Figure 2A). The content of CTX-I in the serum of mice in the IR group was significantly increased compared with that in the Control group, while the content of PINP was significantly decreased (*p* < 0.0001). This result suggests that bone resorption in mice in the IR group was enhanced, bone formation was relatively insufficient, and the overall bone metabolic balance shifted towards bone loss [14]. After the intervention of PGs, the decrease in CTX-I and the increase in PINP in the serum of mice compared with the IR group indicated that PGs could improve the abnormalities of bone metabolism, inhibit bone resorption, and promote bone formation.

Our histological observation by TRAP staining (Figure 2B) showed that the activity of osteoclasts in the IR group was significantly increased (*p* < 0.05), suggesting that radiation can induce abnormal activation of osteoclasts with enhanced bone resorption. And, after the intervention of PGs was given, the activity of osteoclasts in the femur of the IR + PGs group decreased significantly (*p* < 0.05) compared with that of the IR group. The staining results were highly consistent with the trend of changes in serum ELISA results (CTX-I), indicating that PGs can effectively inhibit the radiation-induced overactive state of osteoclasts, thereby reducing bone resorption damage and having a clear protective effect on the structure and function of bone tissue.

The three-dimensional reconstruction results of micro-computed tomography (micro-CT) of the femur further morphologically confirmed radiation-induced bone injury (Figure 2C,D). Compared with the Control group, parameters such as bone volume fraction (BV/TV), trabecular Connectivity, trabecular connection density (Conn.D), and bone mineral density (BMD) in the IR group decreased significantly. The structural pattern index (SMI) and trabecular bone pattern factor (Tb. Pf) were significantly increased (*p* < 0.01), and significant degeneration and reduction in bone mass occurred in the trabecular bone structure of the femur in the IR group of mice. After PGs intervention, BV/TV, Connectivity, Conn.D, and BMD significantly increased, and the health status of bones was significantly improved. The decrease in Tb. Pf and SMI indicates that the rod-shaped trabeculae are transforming into plate-shaped trabeculae, and the trabecular structure is being repaired and reconstructed, suggesting that PGs can effectively reverse the bone loss and bone microstructure damage caused by radiation.

### 2.3. Single-Cell Transcriptomics Reveals Cellular Landscape of the Bone Marrow Microenvironment

**Figure 3:**
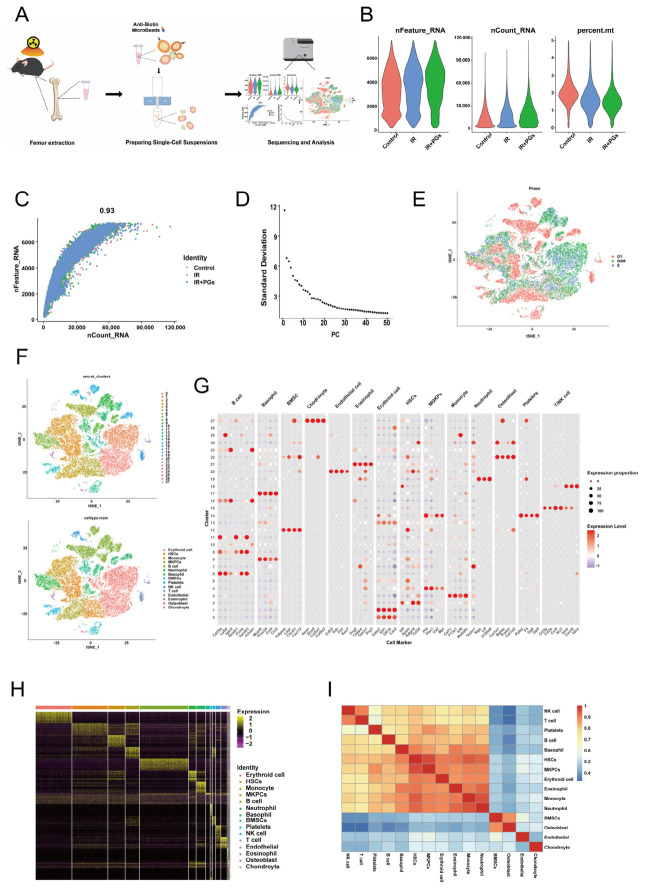
Construction of a panoramic single-cell atlas of the bone marrow microenvironment. (**A**) Schematic workflow for the preparation of single-cell suspensions from mouse bone marrow. Bone marrow cells were harvested from bilateral femurs, enriched for cells using magnetic bead-based sorting, and subsequently subjected to single-cell RNA sequencing. (**B**) Quality control metrics of the transcriptomic data. Cells were filtered based on the following criteria: number of detected genes (nFeature_RNA) between 200 and 7500, and mitochondrial gene percentage (percent.mt) below 5%. (**C**) Scatter plot showing the correlation between the number of detected genes (nFeature_RNA) and total UMI counts (nCount_RNA) per cell. (**D**) Elbow plot derived from principal component analysis (PCA), used to determine the optimal number of principal components for downstream clustering analysis. (**E**) Visualization of cell cycle phase distribution across the dataset (G1, S, and G2M phases). (**F**) t-SNE projection of all cells after dimensionality reduction and clustering, colored by identified cell populations. HSCs: hematopoietic stem cells; MGKPs: megakaryocyte progenitors. (**G**) Bubble plot showing the expression distribution of canonical marker genes used to define each cell cluster. (**H**) Heatmap displaying the top 50 most highly expressed genes within each defined cell cluster. (**I**) Heatmap depicting the transcriptional similarity between different cell clusters based on their gene expression profiles.

In order to systematically reveal the long-term effects of radiation-delayed injury on the bone marrow microenvironment and the intervening role of PGs, we performed single-cell transcriptome sequencing (scRNA-seq) on femoral bone marrow samples from the Control, IR, and IR + PGs groups (Figure 3A). After strict quality control (QC), we excluded cells with ≥5% of mitochondrial genes, retained cells with 200–7500 genes (Figure 3B), and finally obtained 39,500 high-quality single-cell transcriptome data for subsequent analysis. The correlation of gene expression between different experimental batches was as high as 0.93 (Figure 3C), indicating that the experiment was stable and the data quality was reliable.

The expression matrix after QC was normalized, and 2000 highly variable genes were identified for downstream analysis. Subsequently, we performed linear scaling (Scaling) and principal component analysis (PCA) on the data. Based on the elbow plot analysis of principal component contributions [15], we selected the top 40 principal components (PCs) for nonlinear downscaling and clustering analysis (Figure 3D). Meanwhile, when normalizing the data, we found that the majority of the cells (Figure 3E) were in the post-DNA replication ready-to-divide (G2 phase) and mitotic phases (M phase), and a large number of “cell cycle-related genes” would be specifically expressed or highly expressed in this phase, thus affecting our subsequent analysis, so we corrected for the cell cycle to reduce the errors caused by the cell cycle. There were 28 cell subpopulations at a resolution of 0.9, which were visualized using t-SNE random neighbor embedding (Figure 3F). By reviewing the literature to identify cell marker genes [16,17,18], we performed a systematic cell type identification of these 28 cell clusters, which resulted in a clear outline of the complex composition of the bone marrow microenvironment, which consists of 9 erythroid and hematopoietic-associated cell subpopulations (Clusters 0, 1, 2, 4, 5, 10, 13, 14, and 26), 14 immune cell subpopulations (including T-cells, B-cells, myeloid cells, etc.; Clusters 3, 6, 7, 8, 9, 11, 15, 16, 17, 18, 19, 21, 23, and 25), 1 endothelial cell subpopulation (Cluster 20), and 4 bone-associated cell subpopulations (bone marrow mesenchymal cells, osteoblasts, and chondrocytes; Clusters 12, 24, 22, and 27). Erythroid and immune cell markers differed from those of conventional mature hematopoietic cells (Figure 3G).

The reliability of our cell clustering results was validated in multiple ways. First, the accuracy of this classification was supported by the heatmap [19] representing genes characteristically expressed in each cluster, showing clear cluster-specific gene expression patterns (Figure 3H). Secondly, the heatmap of correlation between cell subgroups showed a significant chunked diagonal structure (Figure 3I), suggesting that cells within the same cluster have highly consistent transcriptome characteristics, while there are obvious differences between different clusters. Together, these results confirm the reliability and biological significance of our constructed single-cell atlas of the bone marrow microenvironment.

### 2.4. Subdivision of BMSCs and Osteoblast Subpopulations

**Figure 4:**
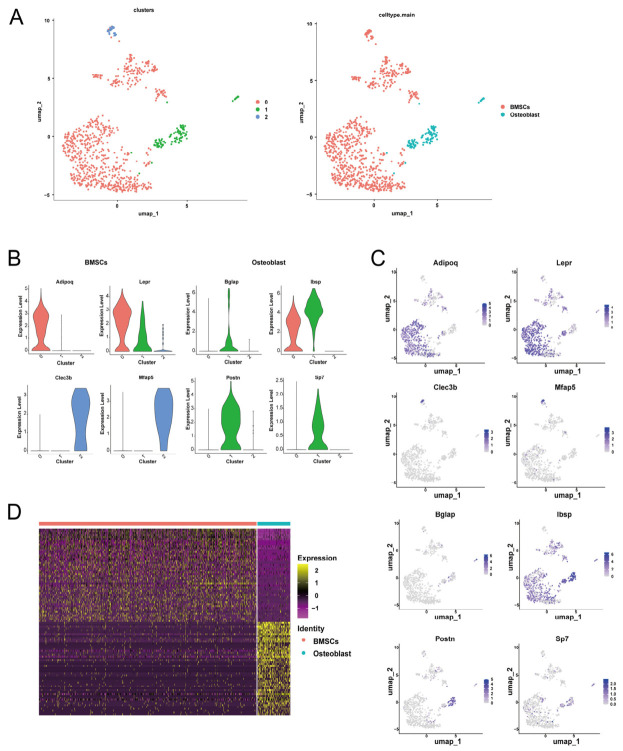
Secondary clustering analysis of bone-related cell populations. (**A**) UMAP visualization of bone-related cells after secondary clustering, colored by cell clusters. (**B**) Violin plots showing the expression of marker genes specific to osteoblast and mesenchymal progenitor cell subpopulations. (**C**) UMAP feature plots illustrating the expression distribution of marker genes in osteoblasts and BMSCs. (**D**) Heatmap of the top 50 most highly expressed genes across osteoblast and BMSCs subpopulations.

In order to deeply explore the effects of radiation on the osteogenic and matrix microenvironment, a key component of bone metabolism, we performed a secondary clustering analysis for bone marrow mesenchymal stem cells (BMSCs; Clusters 12 and 24) and osteoblast populations (Cluster 22). Based on the pre-screening, we reconstructed the subpopulations of these cells (Figure 4A). The analysis showed (Figure 4B,C) that Cluster 0 and Cluster 2 (BMSCs) highly expressed signature genes such as *Adipoq*, *Lepr*, *Mfap5*, and *Clec3b* [20,21], which are characterized as adipose stromal and stromal progenitor cells. In contrast, Cluster 1 (osteoblast) specifically highly expresses the mature osteoblast markers *Bglap*, *Ibsp*, *Sp7*, and *Postn* [22]. As shown in the gene characterization heatmap (Figure 4D), these two major cell populations are fundamentally different in their gene expression profiles, clearly defining their respective unique molecular identities and functional statuses and laying a reliable cellular categorization foundation for subsequent exploration of the specific effects of radiation on their functions.

### 2.5. PGs Promote the Recovery of Osteogenic Differentiation Function of BMSCs

**Figure 5:**
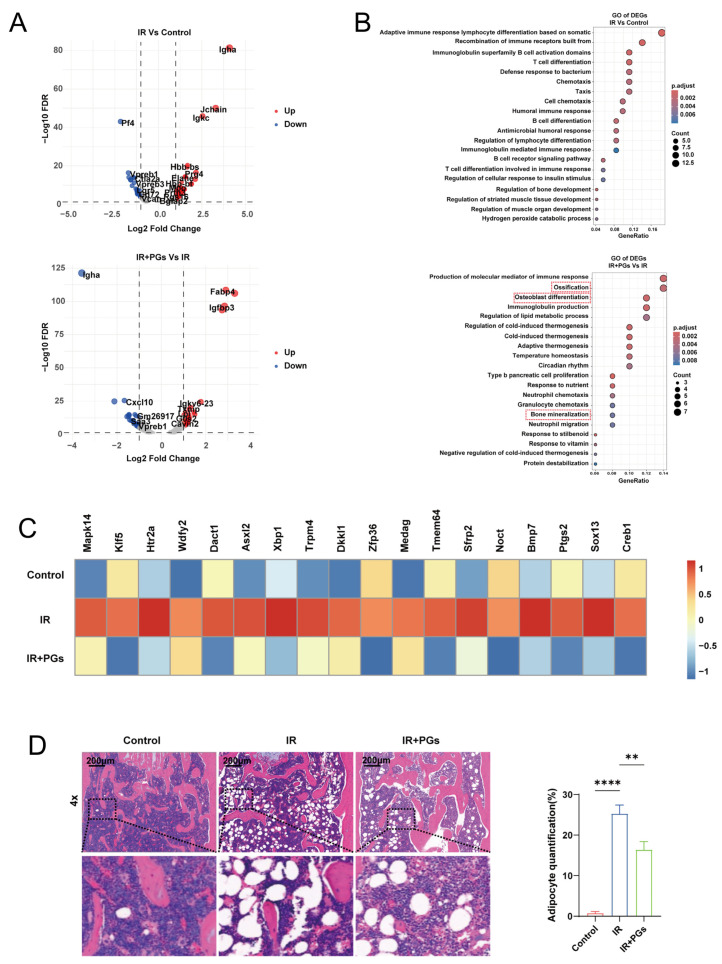
PGs inhibit adipogenic differentiation of BMSCs. (**A**) Volcano plot of differentially expressed genes (DEGs) between groups (upregulated: log2FC ≥ 1; downregulated: log2FC < −1; FDR < 0.05). (**B**) Top 20 enriched biological process terms from GO analysis (*p* < 0.05). (**C**) Heatmap showing the expression of genes associated with positive regulation of adipocyte differentiation (GO:0045600). (**D**) HE staining of femoral sections showing that PGs reduce radiation-induced adipocyte accumulation; quantification of the area proportion of “vacuole-like” structures (adipocytes) within a fixed region are shown. Data are presented as mean ± SD. ** *p* < 0.01, **** *p* < 0.0001.

We performed differential expression gene (DEG) analysis between groups for BMSCs (Figure 5A). Compared with the Control group, the IR group identified 79 DEGs (FDR < 0.05). The IR + PGs group identified 54 DEGs compared to the IR group (FDR < 0.05). Further GO functional enrichment analysis (Figure 5B) showed that the differentially expressed genes in the IR + PGs group were significantly enriched in the biological processes of mineralization and osteoblast differentiation compared with those in the IR group, whereas the differentially expressed genes in the IR group were mainly enriched in a variety of immune and inflammatory response-related pathways, suggesting that radiation disrupts the function of BMSCs. Radiation may have disturbed the normal differentiation trajectory of BMSCs and inhibited their osteogenic differentiation ability. Furthermore, the analysis revealed that the set of genes related to “positive adipocyte differentiation” (GO:0045600) in the IR group showed a significantly high expression (Figure 5C), which was reversed by the treatment of PGs.

This result was further confirmed by HE staining of mouse femurs (Figure 5D), which showed significant pathological changes in the structure of the bone marrow cavity in the femurs of the IR group compared with the Control group, with a significant reduction in hematopoietic tissue, which was replaced by a large number of adipocyte aggregates, which appeared as numerous irregularly shaped “vacuolated” areas in the stained sections. This phenomenon reveals the pathological process of radiation-induced bone marrow adipocytosis, indicating that the homeostasis of the bone marrow microenvironment has been severely disrupted, and the balance of osteoblastic–adipogenic differentiation is tilted towards adipogenesis. After intervention with PGs, the pathologic state of femoral bone marrow was significantly improved. Compared with the IR group, the bone marrow cavity of the IR + PGs group showed a significant reduction in vacuolated structures (*p* < 0.01), preservation of hematopoietic tissue space, and a rebound in overall cell density. This result indicated that PGs could effectively inhibit the process of radiation-induced bone marrow steatosis and maintain the stability of the bone marrow microenvironment to a certain extent.

### 2.6. High Expression of Grem1 in Osteoblasts of Mice with Radiologic Bone Injury

**Figure 6:**
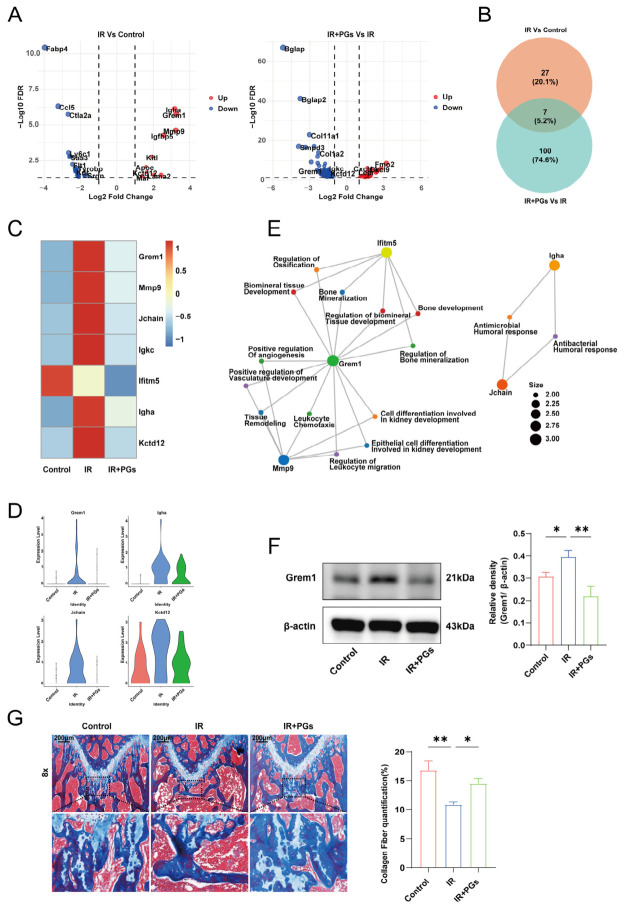
PGs suppress Grem1 expression in osteoblasts. (**A**) Volcano plot of differentially expressed genes (DEGs) in osteoblasts between groups (upregulated: log2FC ≥ 1; downregulated: log2FC < −1; FDR < 0.05). (**B**) Venn diagram illustrating the overlap of DEGs among different groups. (**C**,**D**) Heatmap and violin plots showing the expression patterns of commonly differentially expressed genes across groups. (**E**) GO enrichment network analysis of the common DEGs, indicating that Grem1 is significantly associated with bone mineralization and skeletal development. (**F**) Western blot analysis and quantitative densitometry of Grem1 expression in femoral tissue from mice (*n* = 3). (**G**) Masson’s trichrome staining showing that PGs treatment promotes collagen fiber formation and deposition in the femur. Data are presented as mean ± SD. * *p* < 0.05, ** *p* < 0.01.

To investigate the changes in osteoblast function in radiological bone injury and the intervention mechanism of Cistanche phenylethanol glycosides, we analyzed the osteoblast populations for differentially expressed genes (DEGs) between groups (Figure 6A). IR group vs. Control group: a total of 34 DEGs were identified (FDR < 0.05), of which 11 were upregulated and 23 were downregulated in gene expression. IR + PGs group vs. IR group: a total of 107 DEGs (FDR < 0.05) were identified, of which 32 genes were upregulated and 72 were downregulated, suggesting that radiation and PGs interventions triggered extensive transcriptome reprogramming.

To further screen out the core genes associated with PGs for repairing radiation-induced bone injury, we took the above two groups of differential gene lists for intersection analysis and obtained a total of seven shared DEGs (Figure 6B). The expression of *Grem1*, *Mmp9*, *Jchain*, *Ighc*, *Igha*, and *Kctd12* was increased in the IR group compared to the Control group, while the expression of *Ifitm5* gene was decreased. The expression trends of these seven genes were changed after intervention with PGs (Figure 6C,D). Specifically, six genes (*Grem1*, *Mmp9*, *Jchain*, *Ighc*, *Igha*, and *Kctd12*) that were upregulated in the IR group were significantly decreased in the IR + PGs group, and this global expression reversal strongly suggests that PGs can effectively antagonize radiation-induced transcriptome disruption in osteoblasts.

GO enrichment analysis of the seven intersecting genes revealed that *Grem1*, a key antagonist of the bone morphogenetic protein (BMP) signaling pathway, was of particular interest. The expression of this gene was significantly upregulated in IR osteoblasts, whereas its expression was significantly suppressed in the IR + PGs group. GO analysis suggested that *Grem1* was enriched for biological processes such as bone mineralization and bone development (Figure 6E).

We performed protein extraction of mouse femur tissue samples and detected the expression level of Grem1 protein by Western blot technique (Figure 6F). The results showed that the expression of Grem1 protein in the IR group was significantly upregulated compared to the expression in the Control group (*p* < 0.05), while its expression in the IR + PGs group showed a significant decrease compared to the IR group (*p* < 0.01). Meanwhile, by Masson staining for histologic evaluation (Figure 6G), we observed a significant decrease in new bone in the IR group and a significant increase in new bone in the PGs group. The surface of bone trabeculae in the IR + PGs group showed distinct blue collagen deposition bands that were more organized compared to the IR group. Additionally, newborn, mineralizing bone tissue was visible in these collagen matrices, which clearly demonstrated that PGs not only provided an orderly collagenous structural scaffold for bone repair but also directly contributed to the subsequent mineralization process, demonstrating a clear bone tissue-guided regeneration ability.

Given the central facilitating role played by the BMP signaling pathway in the regulation of osteogenic differentiation and bone formation [23], Grem1, as a classical secreted antagonist of the BMP pathway, competitively binds to BMP ligands, which in turn blocks the binding of BMP to receptor complexes, impedes its translocation to the nucleus and its binding to the promoters of osteogenesis-related target genes [24], and thereby inhibits the normal initiation and operation. Combined with the abnormally high expression of Grem1 protein in post-irradiation osteoblasts observed in this study, we hypothesize that Grem1 is likely to play a key negative regulatory role in the pathologic process of radiological bone injury.

### 2.7. PGs Inhibit Grem1 Protein in MC3T3-E1 Cells

**Figure 7:**
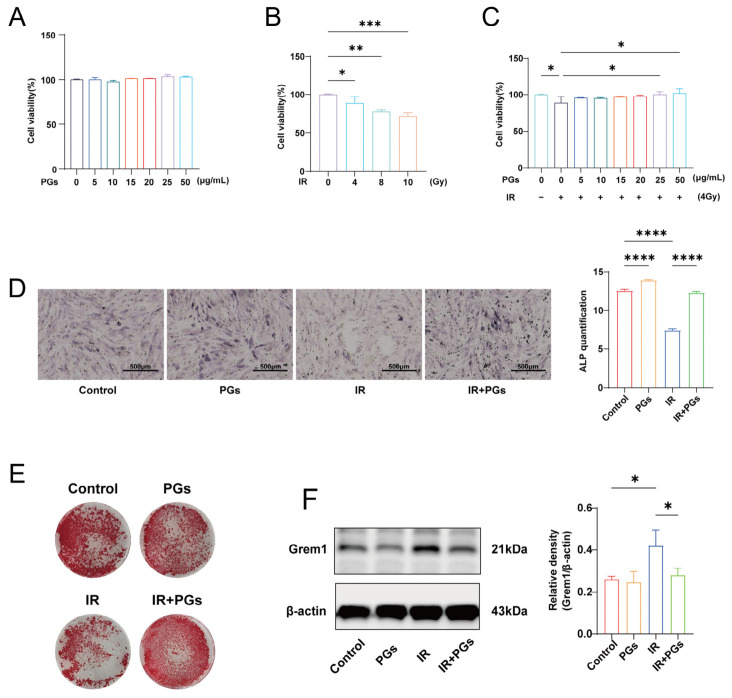
PGs promote osteogenic differentiation and mineralization of MC3T3-E1 cells after irradiation. (**A**) Effects of PGs on the viability of MC3T3-E1 cells, showing no cytotoxicity within the concentration range of 5–50 μg/mL (*n* = 6). (**B**) Effects of irradiation on cell viability, demonstrating a dose-dependent decrease in viability (*n* = 6). (**C**) PGs treatment significantly reverses the reduction in MC3T3-E1 cell viability induced by 4 Gy irradiation (*n* = 6). (**D**) Alkaline phosphatase (ALP) staining and quantitative analysis after 7 days of osteogenic induction, showing that PGs markedly restore early osteogenic capacity in irradiated MC3T3-E1 cells (*n* = 3). (**E**) Alizarin Red S (ARS) staining after 21 days of osteogenic induction, indicating that PGs significantly rescue radiation-impaired mineralization capacity. (**F**) Western blot analysis showing that PGs suppress radiation-induced Grem1 protein expression in MC3T3-E1 cells (*n* = 3). Data are presented as mean ± SD. * *p* < 0.05, ** *p* < 0.01, *** *p* < 0.001, **** *p* < 0.0001.

We used MC3T3-E1 cells for in vitro validation. CCK-8 assay showed that PGs had no significant inhibitory effect on cell viability in the concentration range of 5–50 μg/mL (Figure 7A). To model radiation damage, we examined the effects of different doses of radiation on cell viability (Figure 7B) and found that cell viability was negatively correlated with the radiation dose; the cell viability was about 80% after 4 Gy irradiation, and therefore, this dose was selected for subsequent experiments. Under this condition, 25 μg/mL of PGs significantly restored the radiation-induced decrease in cell viability (Figure 7C).

We performed ALP staining and alizarin red staining analysis. ALP is a key marker enzyme for early osteogenic differentiation, and its activity can visually reflect the early osteogenic status of the cells, whereas alizarin red staining was used to evaluate the late mineralization capacity of the cells by binding specifically to calcium nodules [25,26]. The results of ALP staining of MC3T3-E1 cells at 7 days after osteogenic induction showed that the ALP activity in the IR group was significantly decreased (*p* < 0.0001), indicating that radiation severely impaired the early osteogenic ability of the cells (Figure 7D); the ALP activity in the IR + PGs group was significantly increased (*p* < 0.0001), indicating that PGs could reverse the radiation-induced inhibition of early osteogenesis; at the same time, the ALP activity in the PGs group (the cells were not irradiated and only drug intervention was performed) was significantly higher than that in the Control group (*p* < 0.0001), indicating that PGs not only reversed radiation-induced early osteogenic inhibition but also further promoted early osteogenic capacity. After 21 days of osteogenic induction, alizarin red staining showed that the formation of calcium nodules in the IR group was significantly reduced and the mineralization ability was obviously weakened (Figure 7E); the intervention of PGs significantly increased the number and area of calcium nodules, which indicated that it could effectively restore and enhance the late mineralization ability of the cells.

We further validated this at the protein level (Figure 7F). The results showed that irradiation specifically induced a significant upregulation of Grem1 protein expression in MC3T3-E1 cells, whereas after intervention with PGs, the Grem1 expression in the IR + PGs group was significantly decreased compared with that in the IR group. Notably, in the unirradiated PGs group, there was no significant change in Grem1 expression compared with the Control group. The above results were consistent with the in vivo results, indicating that radiation specifically activated Grem1 expression in osteoblasts, and PGs were able to inhibit this abnormal upregulation.

## 3. Discussion

In this study, we systematically evaluated the protective effects of phenylethanoid glycosides (PGs) from *Cistanche tubulosa* against radiation-induced delayed bone injury and explored the underlying mechanisms at multiple levels. Our results show that PGs improve systemic physiological status after irradiation and exert pronounced reparative effects on bone metabolism, bone microarchitecture, and the bone marrow microenvironment. Integrating single-cell transcriptomics with in vivo and in vitro validation, we further identified Gremlin-1 (Grem1), a BMP signaling antagonist, as aberrantly activated in osteoblasts following irradiation. PGs effectively suppressed its expression and promoted osteogenic recovery. These findings suggest that Grem1 may represent a potential molecular target for intervention in radiation-induced bone injury.

In the present study, irradiated mice exhibited body weight loss, reduced grip strength, elevated oxidative stress, and progressive bone loss, consistent with previous reports [27,28,29]. PGs treatment improved body weight and motor performance and significantly alleviated oxidative stress. Peripheral blood indices, notably, were not markedly restored, indicating that PGs may play a partial role, primarily through improving the bone marrow microenvironment. Given that oxidative stress plays a central pathogenic role in radiation-induced damage to bone and bone marrow [30], the antioxidant properties of PGs likely contribute to their early protective effects.

At the level of bone remodeling, radiation-induced enhancement of bone resorption and suppression of bone formation have been widely reported, mainly due to osteoclast overactivation and impaired osteoblast differentiation [31]. Similarly, increased CTX-I, decreased PINP, increased osteoclast activity, and trabecular deterioration were observed after irradiation in this study. PGs intervention inhibited osteoclast overactivation and promoted deposition and mineralization of new bone matrix. Micro-CT analysis further confirmed improvements in bone volume fraction, trabecular connectivity, and bone mineral density. These results indicate that PGs help restore the disrupted balance between bone resorption and bone formation after radiation exposure.

Bone marrow adiposity is recognized as a key pathological feature of late radiation-induced bone injury [32,33]. Previous studies have shown that irradiation drives BMSCs toward adipogenic differentiation while suppressing osteogenesis, thereby impairing the bone marrow microenvironment and limiting bone repair capacity [34,35]. Consistently, HE staining in our study revealed marked accumulation of marrow adipocytes after irradiation, which was significantly attenuated by PGs treatment. Single-cell transcriptomic analysis further demonstrated that PGs restored osteogenic and mineralization-related gene programs in BMSCs while suppressing adipogenic signatures. These findings suggest that PGs contribute to maintenance of bone marrow homeostasis by modulating BMSCs lineage commitment, thereby providing sustained cellular support for osteogenesis.

A key observation of this study is the radiation-induced upregulation of Grem1 in osteoblasts. Grem1 is a classical secreted antagonist of BMP signaling; it binds BMP ligands and prevents their interaction with BMP receptors, thereby suppressing Smad-dependent osteogenic transcriptional programs [36,37], and its overexpression impairs osteoblast differentiation and leads to bone loss [38]. Previous studies have shown that dysregulated Grem1 expression restrains osteoblast differentiation and participates in various bone metabolic disorders [39,40]. Although the upstream signals that drive Grem1 expression in irradiated osteoblasts remain to be clarified, a similar pathway has been reported in other skeletal cells. Interestingly, it has been shown that reactive oxygen species (ROS) activate the NF-κB pathway in chondrocytes to promote the expression of Grem1, which exacerbates the development of osteoarthritis [41]. Whether radiation-induced ROS similarly activates NF-κB to upregulate Grem1 in osteoblasts is an important question for future investigation. In our study, Grem1 was markedly elevated in osteoblasts after irradiation at both the transcript and protein levels, whereas PGs treatment significantly reduced its expression. In vitro experiments using MC3T3-E1 cells further confirmed that radiation-induced Grem1 upregulation was accompanied by impaired osteogenic differentiation, which was partially restored by PGs. These results suggest that aberrant activation of Grem1 contributes to radiation-associated osteogenic suppression and that PGs may promote osteoblast function by relieving Grem1-mediated inhibition of BMP signaling. Given the essential role of BMP signaling in bone formation [42], targeting the Grem1-BMP axis may represent an effective strategy for intervention in radiation-induced bone injury.

Several limitations of this study should be acknowledged. Although multi-omics analyses and functional experiments support a critical role for Grem1 in mediating the osteoprotective effects of PGs, direct causal validation using Grem1 knockdown or overexpression models has not yet been performed. Furthermore, the limited effect of PGs on hematological recovery indicates the need to explore combination therapies, possibly with hematopoietic growth factors, to achieve comprehensive rehabilitation of the bone marrow microenvironment. Future studies will therefore pursue two main directions: first, genetic manipulation of Grem1 or specific modulation of BMP signaling will be employed to clarify the necessity and specificity of the PGs–Grem1–BMP axis in radiation-induced bone injury; second, a comparative analysis of pure PGs components, such as echinacoside and acteoside, will be performed to identify the most active compound and to elucidate the precise molecular mechanism underlying Grem1 suppression.

## 4. Materials and Methods

### 4.1. Experimental Animals, Grouping, and Treatment

Seven-week-old male C57BL/6J mice (21 ± 1 g) were purchased from Beijing Weitonglihua Laboratory Animal Technology Co., Ltd. (Beijing, China), and acclimatized in an SPF-grade animal facility for one week. The mice were randomly divided into three groups: blank control group (Control), irradiated group (IR), and post-irradiation Cistanche phenylethanoid glycoside intervention group (IR + PGs). There were 20 in each group. The experimental period was 8 weeks, and the total dose of 10 Gy was irradiated in two sessions. In order to avoid the uneven radiation dose caused by the activity of the mice during irradiation, we used a special radiation box (the box has ventilation holes to ensure normal breathing of the mice) to immobilize the mice and put the mice inside the box to reduce their activity. Mice in the Control group were placed in the immobilization device without irradiation. Mice in the IR and IR + PGs groups were fixed in the irradiation room and received total body irradiation. The total dose of the first irradiation was 5 Gy (dose rate: 85.04 R/min), using a 60Co radiation source provided by the Beijing Institute of Radiation Medicine. Four weeks after the first irradiation, a second irradiation with the same dose was administered.

After completion of the second irradiation, mice in the IR + PGs group were gavaged daily with 0.1 mL (the concentration of the drug solution was adjusted according to the body weight of the mice) aqueous solution of Cistanche phenylethanoid glycosides (Kanion Pharmaceutical, 231008, Lianyungang, China. 0.38 g of echinacoside per 1 g of dry product, and 0.75 g of total PGs) at a dose of 150 mg/kg. Mice in the Control and IR groups were gavaged with an equal volume of saline for 4 weeks. At the end of the intervention, we anesthetized the mice and collected blood using the eye removal method; 150 μL of whole blood was collected in a 1.5 mL anticoagulated tube for complete blood count analysis. Afterwards, about 1 mL of blood was collected in a non-anticoagulated 1.5 mL centrifuge tube and left at room temperature for 1 h. After clotting, the blood was centrifuged at 4 °C, 3000× *g*, for 15 min, and at the end of centrifugation, the upper layer of serum was aspirated, placed in new 1.5 mL centrifuge tubes, and frozen at −80 °C for subsequent experimental testing. At the end of blood sampling, the mice were executed and the femurs were collected. Femurs were either fixed in 4% paraformaldehyde or stored at −80 °C for further experiments. Any remaining femurs were used for preparation of bone marrow single-cell suspensions.

### 4.2. Micro-Computed Tomography

Micro-computed tomography (micro-CT, PINGSENG Healthcare (Kunshan) Inc., VNC-102, Kunshan, China) was used for three-dimensional structural analysis of fixed femoral samples. Scanning parameters were set at 80 kV tube voltage and 0.08 mA tube current. After fixation, samples were gently blotted to remove surface liquid and placed on the scanning stage. Raw data were reconstructed using Recon software and analyzed using Avatar software for bone morphometric evaluation. The region of interest (ROI) was defined as trabecular bone beginning 0.5 mm below the growth plate and extending 1.5 mm distally. Trabecular bone parameters within the ROI were extracted and exported for statistical analysis.

### 4.3. Hematoxylin–Eosin (HE) Staining

Bilateral femurs were isolated, and surrounding soft tissues were carefully removed. Samples were fixed in 4% paraformaldehyde for 48 h and then decalcified in 10% EDTA solution (pH 7.4) at 4 °C until complete decalcification was achieved. The decalcification solution was replaced daily. After decalcification, tissues were dehydrated through graded ethanol, cleared in xylene, embedded in paraffin, and sectioned along the sagittal plane. Sections were baked at 60 °C for 2 h and stained with hematoxylin–eosin. Pathological images were scanned for analysis. Image J (version 1.54g) was used to calculate the proportion of vacuoles (adipocytes) in the fixed area at the same location under 1 mm of the growth plate.

### 4.4. Tartrate-Resistant Acid Phosphatase (TRAP) Staining

Decalcified paraffin sections were deparaffinized in xylene and rehydrated through graded ethanol to distilled water. Sections were incubated in distilled water preheated to 37 °C for 15 h, then alkalized for 1 h, and rinsed. Freshly prepared TRAP incubation solution (Servicebio, G1050, Wuhan, China) was added, and sections were incubated at 37 °C for 20 min. After staining, nuclei were counterstained with hematoxylin for approximately 15 s, differentiated, and blued (sections were immersed into the reverse blue solution for 10 s, then removed, and washed with water). Sections were dehydrated, cleared, sealed with neutral gum, and scanned. Image J was used to calculate the percentage of red areas (osteoclasts) within the fixed area at the same location of the growth plate.

### 4.5. Masson Staining

After deparaffinization and rehydration, Masson trichrome staining (Servicebio, G1006, Wuhan, China) was performed according to the manufacturer’s instructions. Sections were fixed in Masson A solution at 37 °C overnight, followed by further incubation at 37 °C for 30 min. Nuclei were counterstained with freshly mixed Masson B and C solutions. After differentiation, sections were stained sequentially with Masson D, E, and F solutions. Finally, sections were dehydrated, cleared, sealed with neutral gum, and scanned. Image J was used to calculate the percentage of blue-green area (new bone) within the fixed area at the same location under the growth plate.

### 4.6. Preparation of Femoral Single-Cell Suspension

After sacrifice, mice were disinfected with 75% ethanol. Femurs and tibias were isolated in 199 medium containing 2% FBS, and soft tissues were removed. Bones were processed using a three-step enzymatic digestion protocol. Epiphyses were separated and digested with 1 mg/mL STEMxyme 1 and Dispase II at 37 °C for 45 min. The diaphysis was cut into small fragments and digested with the same enzyme solution at 37 °C for 30 min. Bone marrow contents were flushed out, centrifuged, and digested again for 30 min. All digestion solutions were combined, filtered through a 70 μm filter, and centrifuged at 1500× *g* for 5 min. Red blood cells were lysed using ACK buffer. Cells were washed, filtered through a 40 μm filter, and resuspended in 199 medium with 2% FBS, and viability was assessed by trypan blue staining.

Cells were adjusted to 2.5 × 10^8^ cells/mL. Biotin-labeled antibody cocktail was added and incubated at 4 °C for 15 min. Anti-biotin magnetic beads (Miltenyi Biotec, Lineage Cell Depletion Kit, mouse, 130-110-470, Bergisch Gladbach, Rhineland, Germany) were then added and incubated at 4 °C for 20 min. Cells were washed, resuspended, and applied to magnetic sorting columns. The effluent of the cells (which includes hematopoietic stem progenitor cells, bone marrow mesenchymal stromal cells, or cells that do not express conventional markers of mature hematopoietic cells) was collected, centrifuged, and used for subsequent single-cell sequencing.

### 4.7. Single-Cell Transcriptome Data Analysis

The single-cell suspension was encapsulated into water-in-oil droplets; lysed; and then sequentially subjected to reverse transcription, cDNA amplification, and library construction. Single-cell RNA sequencing libraries were prepared according to the manufacturer’s protocol. Libraries were sequenced on a NovaSeq 6000 platform (Illumina, San Diego, CA, USA), and raw sequencing data were processed using Cell Ranger (version 7.0.1) for sample splitting, comparison to the mouse reference genome, and generation of gene-barcode matrices. Raw sequencing data were processed using Seurat V5 (version 5.1.0) in R (version 4.4.1). Cells expressing 200–7500 genes and <5% mitochondrial genes were retained. Cell-cycle scores were calculated and regressed out to minimize cell-cycle effects. Data integration across samples was performed using Harmony based on the first 40 principal components. Clustering resolution was set at 0.9 based on clustree evaluation, and visualization was performed using t-distributed Stochastic Neighbor Embedding (t-SNE) or Uniform Manifold Approximation and Projection (UMAP). Cluster annotation was conducted using marker genes identified by FindAllMarkers, referencing CellMarker 2.0 and PanglaoDB databases.

### 4.8. Differential Gene Analysis

Osteoblast and bone marrow mesenchymal stem cell subpopulations were extracted from the integrated dataset. Differential gene expression analysis was performed using edgeR (version 4.2.2). Screening criteria were FDR < 0.05 and |log2FC| > 1. Gene ontology (GO) enrichment analysis was performed using the enrichGO function in clusterProfiler (version 4.12.6) to explore biological functions and signaling pathways.

### 4.9. MC3T3-E1 Cell Culture and Processing

MC3T3-E1 cells (Procell, CL-0378, Wuhan, China) were cultured in MEM-α medium containing 10% fetal bovine serum and 1% penicillin-streptomycin. Irradiation conditions were determined using CCK-8 assays. The cells were irradiated with 4 Gy at 2 m from a cobalt source. The groups included Control, IR, PGs (without irradiation), and IR + PGs. After 24 h of irradiation, the PGs (without irradiation) and IR + PGs groups were cultured with medium containing 25 μg/mL PGs. All groups were incubated for 48 h before further analyses.

### 4.10. Alizarin Red Staining

MC3T3-E1 cells were seeded in 12-well plates at 5 × 10^4^ cells/well. After irradiation and drug treatment, osteogenic induction medium (Procell, PD-033, Wuhan, China) was applied and replaced every two days. After 21 days, the cells were fixed with 4% paraformaldehyde and stained with alizarin red solution (Beyotime, C0148S, Shanghai, China). Images were acquired.

### 4.11. Alkaline Phosphatase (ALP) Staining

After 7 days of osteogenic induction, MC3T3-E1 cells were stained using BCIP/NBT solution (Beyotime, C3206, Shanghai, China). Images were captured after color development.

### 4.12. Western Blot

Femur tissue or MC3T3-E1 cells were lysed in RIPA buffer with protease and phosphatase inhibitors. Protein concentrations were determined using BCA assay. Proteins were separated by 10% SDS-PAGE and transferred to membranes. Membranes were blocked (5% skimmed milk closed at room temperature for 1.5 h) and incubated overnight at 4 °C with anti-Grem1 antibody (Proteintech, 18024-1-AP, 1:400) and anti-β-actin antibody (1:1000), followed by incubation with secondary antibodies (Proteintech, SA00001-2, 1:5000) at room temperature. Signals were detected using ImageQuant LAS 500 system and quantified by Image J.

### 4.13. ELISA

Mouse CAT, CTX-I, and PINP levels were measured using ELISA kits (Jiangsu Meimian Industrial Co., Ltd., MEIMIAN, MM-44125M1, MM-44831M2, and MM-44814M2, Yancheng, China) according to the manufacturer’s instructions.

### 4.14. Statistical Analysis

Data were analyzed using paired *t*-tests when normality and homogeneity of variance were satisfied; otherwise, rank-sum tests were applied. Significance was set at *p* < 0.05. Analyses were performed using GraphPad Prism 9.5.

## 5. Conclusions

In summary, our findings demonstrate that PGs mitigate radiation-induced bone injury through multiple coordinated mechanisms, including attenuation of oxidative stress, restoration of bone remodeling balance, correction of BMSC differentiation bias, and suppression of Grem1-mediated inhibition of osteogenesis. This multi-target mode of action is consistent with the complex pathophysiology of radiation-induced skeletal damage and provides experimental evidence supporting the potential application of Cistanche-derived bioactive compounds in radiation-induced bone injury repair.

## Data Availability

Dataset available from the authors on request.
